# Genome Sequence of *Fusarium* Isolate MYA-4552 from the Midgut of *Anoplophora glabripennis*, an Invasive, Wood-Boring Beetle

**DOI:** 10.1128/genomeA.00544-16

**Published:** 2016-07-21

**Authors:** Joshua R. Herr, Erin D. Scully, Scott M. Geib, Kelli Hoover, John E. Carlson, David M. Geiser

**Affiliations:** a Department of Plant Pathology and Center for Plant Science Innovation, University of Nebraska, Lincoln, Nebraska, USA; b Stored Product Insect and Engineering Research Unit, USDA, Agricultural Research Service, Center for Grain and Animal Health Research, Manhattan, Kansas, USA; c Tropical Crop and Commodity Protection Research Unit, USDA-ARS Daniel K. Inouye Pacific Basin Agricultural Research Center, Hilo, Hawaii, USA; d Department of Entomology and Center for Chemical Ecology, The Pennsylvania State University, University Park, Pennsylvania, USA; e The Schatz Center for Tree Molecular Genetics, Department of Ecosystem Science and Management, The Pennsylvania State University, University Park, Pennsylvania, USA; f Department of Plant Pathology & Environmental Microbiology, The Pennsylvania State University, University Park, Pennsylvania, USA

## Abstract

The *Fusarium solani* species complex (FSSC) is a clade of environmentally ubiquitous fungi that includes plant, animal, and insect associates. Here, we report the draft genome sequence of the undescribed species FSSC 6 (isolate MYA-4552), housed in the gut of the wood-boring cerambycid beetle *Anoplophora glabripennis*.

## GENOME ANNOUNCEMENT

The *Fusarium solani* species complex (FSSC) is a ubiquitous and functionally diverse group of fungi consisting of plant pathogens (1), human pathogens (2), and insect associates, including nutritional symbionts (3–5). Of the symbionts inhabiting the Asian longhorned beetle (ALB) gut, FSSC 6 has been persistently vertically transmitted in a colony maintained in quarantine at Penn State University, PA, for 16 generations and has been detected in all field-collected specimens in the United States (5, 6). Previous analyses have demonstrated that FSSC 6 can degrade lignocellulose (7), synthesize several essential amino acids (6), and contribute to the synthesis of sterols and other essential nutrients (8). To more thoroughly understand the metabolic capacities of FSSC 6 and its potential roles in ALB digestive physiology, we sequenced and assembled the genome of isolate ATCC MYA-4522 derived from an ALB larva collected in New York (4, 5).

Genomic DNA was extracted from freeze-dried tissue using the QIAamp DNA minikit (Qiagen, Redwood City, CA). Following extraction, genomic DNA (gDNA) concentration and integrity were assessed via agarose gel electrophoresis and using a NanoDrop ND-8000 UV-Vis spectrophotometer (Thermo Fisher Scientific). Three 454 FLX+ pyrosequencing libraries (Roche, Branford, CT) were prepared using the GS FLX Titanium sequencing kit XLR70, according to the manufacturer’s specifications. The three 454 FLX+ runs provided a total of 7.138 Gb of single-end sequencing data. Low-quality base calls and 454-specific sequencing adapters were removed using Trimmomatic (9). Sequencing errors, assessed as erroneous k-mers, and redundant data over 30× coverage were filtered using the khmer software package (10). A combined filtered data set provided initial assembly drafts using the SPAdes (11), SOAP*denovo* (12), and Velvet (13) genome assembly programs, and contigs from each assembler were combined into scaffolds using Mix (14) and the haploid *Nectria haematococca* (FSSC undescribed species [11]) genome assembly as a guide (15). The final assembly consisted of 103 contigs, with a G+C content of 51.84%, and with a total length of 46.9 Mbp; this length represents 99% of the estimated genome size from k-mer spectral analysis (10). The largest contig (prescaffolding) was 4,887,614 bases in length, and the assembly consisted of an *N*_50_ of 1,729,713 bp, with half of the genome represented by eight contigs. While approximately 91.9% of the genome assembly aligned to the FSSC 11 genome, 200 kbp of small contigs putatively represent supernumerary chromosomal regions. Total reads, as well as small contigs, were mapped using Bowtie 2 (16) to both the genome assembly and to the NCBI reference genome collection and were verified to be of fungal origin. Utilizing gene models optimized for fungi and gene features from previously sequenced *Fusarium* genomes (15, 17), the program MAKER (18) was used to annotate the FSSC 6 MYA-4552 genome. This annotation predicted 13,157 genes represented by open reading frames.

### Nucleotide sequence accession number.

The whole-genome shotgun sequencing project for the FSSC 6 isolate MYA-4552 has been deposited at DDBJ/EMBL/GenBank under the accession no. LWBZ00000000. The version described in this paper is the first version of the assembly.
